# Factors Influencing Monkeypox Vaccination: A Cue to Policy Implementation

**DOI:** 10.1007/s44197-023-00100-9

**Published:** 2023-04-29

**Authors:** Priyobrat Rajkhowa, Viola Savy Dsouza, Rashmi Kharel, K. Cauvery, B. Rashmi Mallya, D. S. Raksha, V. Mrinalini, Preejana Sharma, Sanjay Pattanshetty, Prakash Narayanan, Chandrakant Lahariya, Helmut Brand

**Affiliations:** 1 Department of Health Policy, Prasanna School of Public Health, Manipal Academy of Higher Education (MAHE), Manipal, Karnataka 576104 India; 2 Department of International Health, Care and Public Health Research Institute-CAPHRI, Faculty of Health Medicine and Life Sciences, Maastricht University, Maastricht, The Netherlands; 3 Institute for Global Health, University College London (UCL), London, UK; 4 Department of Global Health Governance, Prasanna School of Public Health, Manipal Academy of Higher Education (MAHE), Manipal, Karnataka 576104 India; 5 Department of Clinical Psychology, Manipal College of Health Professions, Manipal Academy of Higher Education (MAHE), Manipal, Karnataka 576104 India; 6 Department of Psychiatric (Mental Health) Nursing, Manipal College of Nursing, Manipal Academy of Higher Education (MAHE), Manipal, Karnataka 576104 India; 7 Integrated Department of Pediatrics and Community Medicine, Foundation for People-centric Health Systems, New Delhi, 110029 India

**Keywords:** Monkeypox, Mpox, Stigma, Vaccination, Accessibility, Access to vaccination

## Abstract

**Background:**

Following the mpox 2022 outbreak, several high-income countries have developed plans with inclusion criteria for vaccination against the mpox disease. This study was carried out to map the factors influencing mpox vaccination uptake to help address the challenges and increase vaccination confidence.

**Methods:**

This was a study based on Tweet analysis. The VADER, Text Blob, and Flair analyzers were adopted for sentiment analysis. The “Levesque conceptual framework for healthcare access” was adopted to evaluate the factors impacting access and the decision to get mpox vaccination. Consolidated Criteria for Reporting Qualitative Research (COREQ) criteria were adopted.

**Findings:**

A total of 149,133 tweets were extracted between 01/05/2022 and 23/09/2022. Around 1% of the random tweets were used for qualitative analysis. Of the 149,113, tweets were classified as positive, negative and neutral, respectively, by (a) VADER: (55,040) 37.05%, (44,395) 29.89%, and (49,106) 33.06%, (b) TextBlob: (70,900) 47.73%, (22,729) 15.30%, and (54,921) 36.97%, and (c) Flair: (31,389) 21.13%, (117,152) 78.87%, and 0.00%. Sentiment trajectories revealed that communication, stigmatization, accessibility to and availability of vaccines, and concerns about vaccine safety as factors influencing decision-making in the content and flow of tweets.

**Interpretation:**

Twitter is a key surveillance tool for understanding factors influencing decisions and access to mpox vaccination. To address vaccine mistrust and disinformation, a social media-based risk communication plan must be devised. Adopting measures to remove logistical vaccination hurdles is needed. Obtaining fact-based information from credible sources is key to improving public confidence.

## Introduction

Vaccination programmes are beneficial for preventing infections and reducing the costs associated with sickness, morbidity, and mortality [1]. On 23 July 2022, the multi-country mpox outbreak was declared a public health emergency of international concern (PHEIC) by the World Health Organization (WHO) [2]. The ongoing mpox outbreak has been inconclusively connected to the Lesbian, Gay, Bisexual, Transgender, and Queer (LGBTQ +) community, making stigma a significant obstacle. Due to erroneous inaccurate information, the virus has occasionally been mislabeled as a “gay disease”, which caused the mpox disease to be mislabelled as the “LGBTQ + community man disease”, and issues of homophobia and stigmatization arose [3, 4]. It is imperative to acknowledge that the incidence of monkeypox is not limited solely to the demographic previously indicated but also encompasses individuals of white descent, people living with HIV (PLHIV), as well as individuals who identify themselves as gay, bisexual and men who have sex with men (MSM) [5–8]. These challenges about the emergence of mpox concerns pose significant difficulties and complexities for individuals accessing the mpox vaccine. In response to the mpox outbreak, many countries have approved smallpox vaccines as off-label against mpox [9, 10]. Smallpox vaccines for the prioritized affected population are the key strategy for mitigating mpox in humans [10, 11]. However, multiple factors, such as a lack of vaccine confidence, have hampered vaccination uptake [12]. Therefore, reaching the population with a fact-based risk communication strategy is essential to ensure the optimum vaccination [10, 13]. Public health policy relies significantly on the development and analysis of public communications.

The pervasive impact of social media has made available an infinite amount of information about the general population’s opinions. Through various studies, it has been established that Twitter, as a social media platform, provides a valuable and innovative means of comprehending the public’s sentiments, moods, and opinions regarding critical global events [14]. Consequently, data from social media sources can be analyzed to achieve syndromic surveillance, address public health concerns, and shape public perception using web-based information. Additionally, this data can facilitate effective communication among social media users and other platforms capable of combating conspiracy theories [15]. Given the gradual spread of mpox in non-endemic countries and the proliferation of misinformation about the disease, it has become increasingly evident that public responses to mpox can be readily discerned through social media platforms such as Twitter [16]. Even in the case of the COVID-19 pandemic, scientific researchers have utilized Twitter data to monitor the emergence of user concerns, the spread of misinformation, and the general sentiment of the public [17]. This, in turn, has impacted access to vaccination against the disease. To increase vaccination rates and decrease mpox infection, it is essential to comprehend the factors that influence vaccination uptake. Therefore, this study has adopted the available Twitter data and the conceptual framework developed by Lévesque et al. to conceptualise access to healthcare [18]. This study's findings would provide us with a more extensive and nuanced understanding of the public’s perceptions of mpox vaccination on Twitter. Such insights would be invaluable for developing more effective policies and health system responses to epidemic preparedness, even in countries where mpox is non-endemic. Additionally, the study results would aid in creating tailored strategies for increasing access to and uptake of mpox vaccine.

## Materials and Methods

**Fig. 1:**
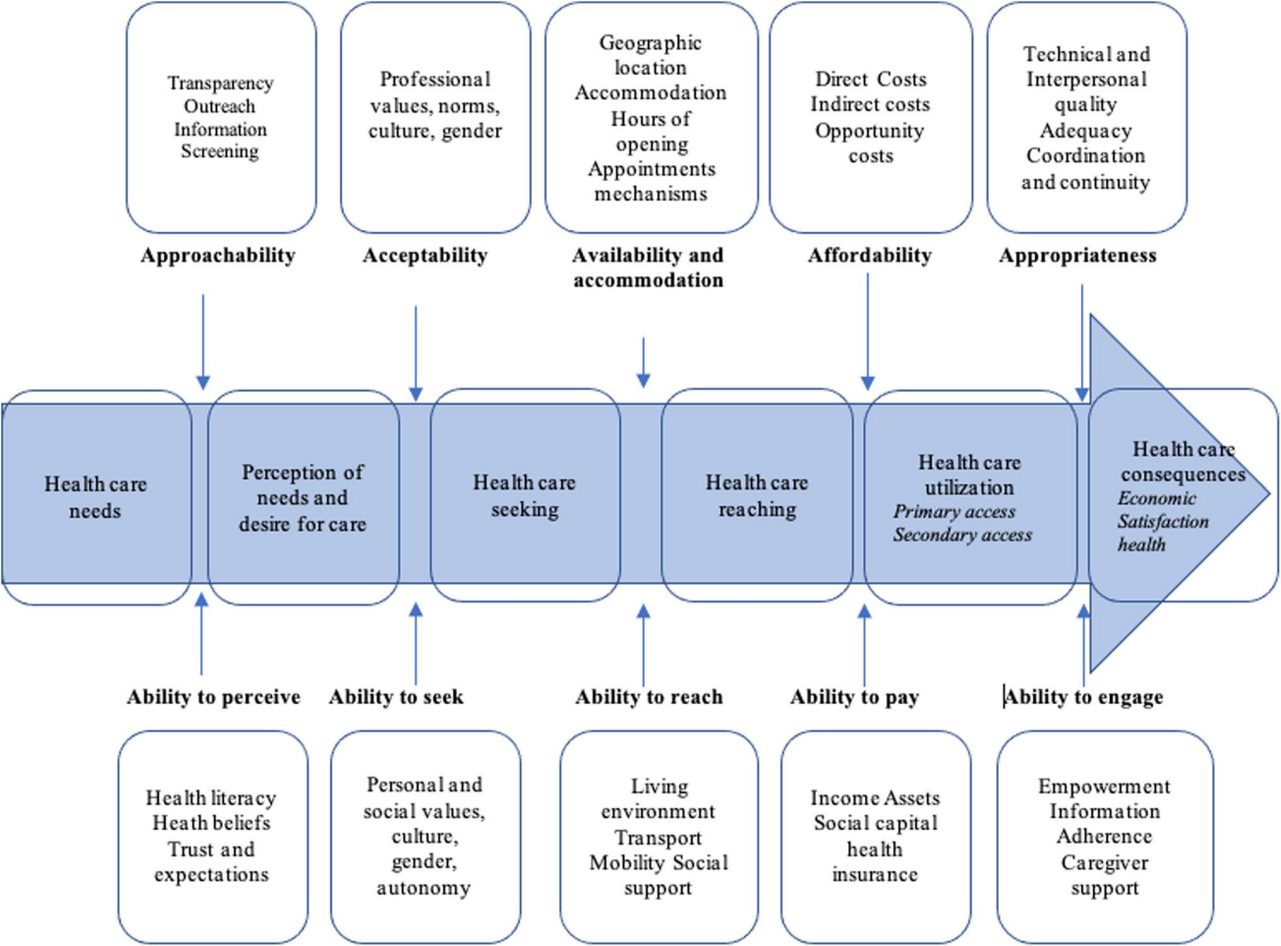
“Levesque conceptual framework for healthcare access” [18]

This study adopts a framework by Lévesque et al.’s conceptualization of access to healthcare to conceptualise and comprehend the factors influencing mpox vaccine uptake [18]. The following factors influence vaccination access are: “approachability, acceptability, availability and accommodation, affordability, and appropriateness, ability to perceive, ability to seek, ability to reach, ability to pay, and ability to engage.” Because this study is based on general population perspectives and aims to comprehend aspects of their abilities to acquire vaccination, we use dimensions/abilities constructs as main themes and highlight issues/concerns from our analysis (Fig. 1, annexure 1).

### Data Acquisition

**Fig. 2:**
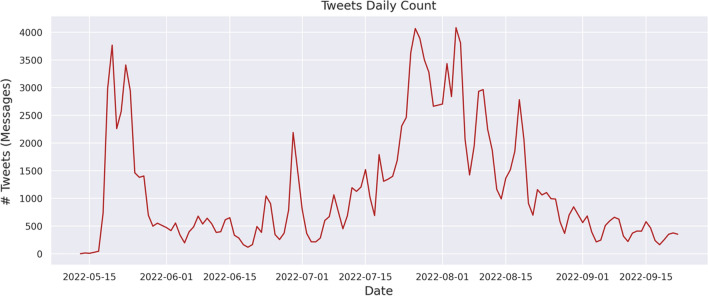
Tweets count over timeline from 01/05/2022 to 23/09/2022

**Fig. 3:**
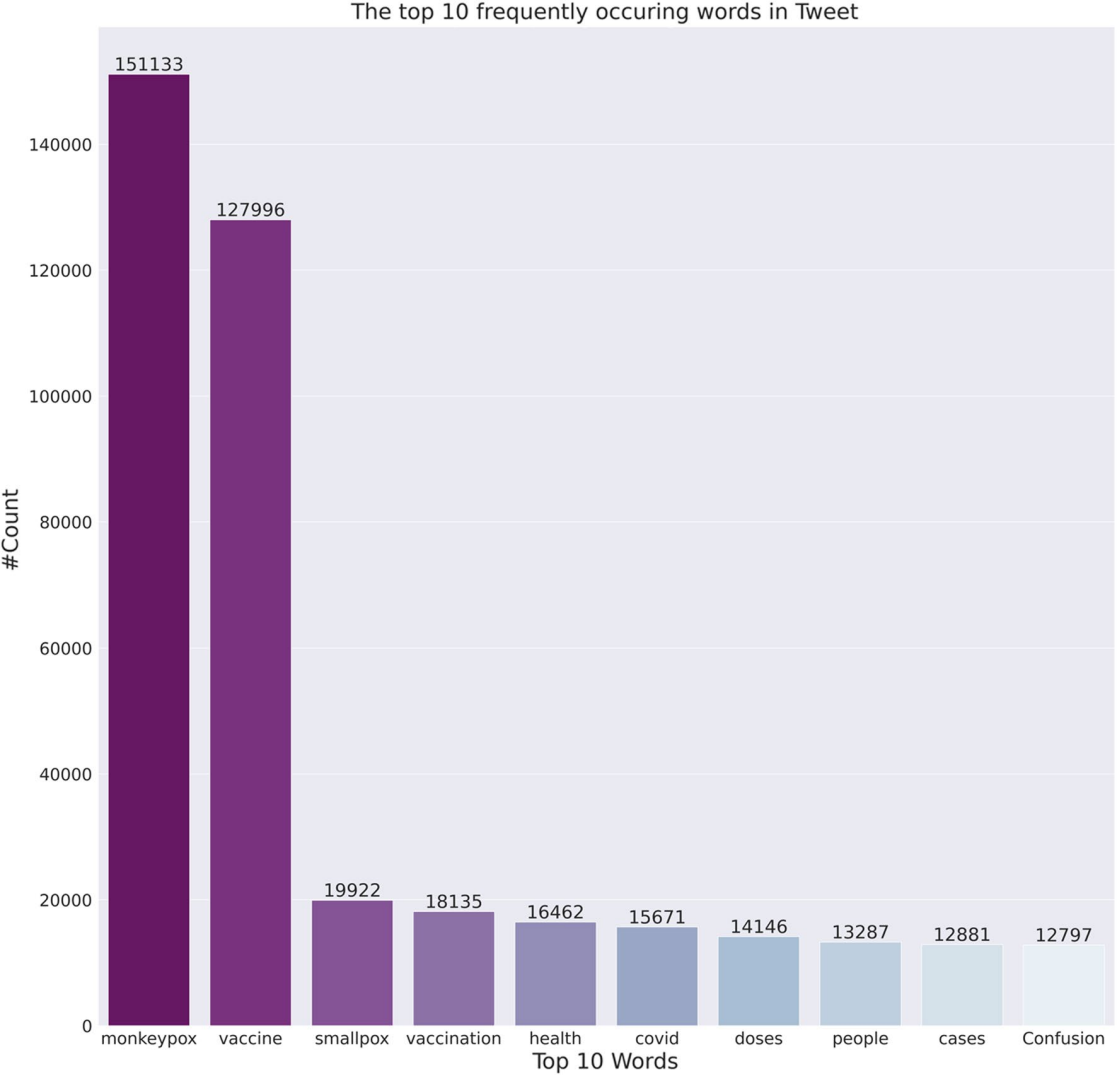
The top 10 occurring words from the tweets

For this study, corresponding (a) tweets containing the keywords “Monkeypox”, “Monkeypoxvirus”, “MPX”, “mpox”, “vaccination”, and “immunisation”, and (b) tweets posted between 01/05/2022 and 23/09/2022 were retrieved. Tweets were obtained using Twitter’s academic research Application Programming Interface (API) v2 [19]. Figure 2 presents a comprehensive illustration of the eligible tweets over time, totaling 152,802. Furthermore, Fig. 3 showcases the frequently recurring words extracted from the aforementioned tweets.

The figure above shows the top 10 words used by the users in tweets. We observe that most of the top terms deal with monkeypox, vaccine, smallpox, vaccination etc.

### Data Pre-processing and Analysis

In this study, Lexicon (or Rule) based models: VADER (Valence Aware Dictionary and Sentiment Reason) and TextBlob and Embedding based model: Flair, were employed for performing sentiment analysis of un-labelled data [20–22]. The following pipeline outlines the workflow of the Sentiment Analysis: data acquisition, pre-processing, and sentiment extraction and classification. Metrics like macro_F1 score (the harmonic mean of the macro-averaged precision and recall) and classification accuracy were utilised to estimate the efficiency of the models. However, the Flair model was excluded from model evaluation as it classifies data as either positive or negative. In contrast, the sample tweets used for model evaluation were annotated for positive, negative and neutral labels. The data analysis was conducted using Python 3.7.14 [3].

The raw tweets were pre-processed using Pandas Library in a series of phases: (i) tweets with the ‘is retweet’ label was marked as duplicate and removed subsequently. Furthermore, additional duplicates were removed based on tweet id and content. (ii) The raw tweets were cleaned as follows: (a) For VADER and TextBlob analysers: tweets were transformed to lower case and hashtags, mentions, web-links, emails, numeric data, and spaces were removed, while for the Flair model, additionally, emojis/emoticons were mapped to their appropriate text, and punctuation and stop-words were omitted [20–22]; (b) tweets containing less than five words were eliminated since certain tweets were left with very few words following the pre-processing stage, which would not contribute to the analysis. (iii) Only tweets in ‘English’ were included. Data obtained in this study were made publicly available by Twitter users and are thus deemed as public domain data. User anonymity was ensured by presenting data in aggregated form. As a result, no additional ethical approval was obtained for this research. This study’s data usage and processing followed Twitter’s Terms of Service and the Developer’s Agreement and Policy.

Followed by sentiment analysis, 1% of all 152,805 tweets were randomly selected for content analysis to strengthen the study’s analytic dependability, confirmability, and trustworthiness. Each coder was provided with 306 tweets. Based on “Levesque conceptual framework for healthcare access”, the coders independently compiled a list of issues concerning factors such as “approachability, acceptability, affordability, appropriateness, availability and accommodation, ability to perceive, ability to seek, ability to reach, ability to pay and ability to engage” [18]. A codebook was created in which the factors influencing vaccination intake served as overarching themes. The particular concerns selected through the open coding process served as coding variables within these themes. Microsoft Excel was used to code the tweets. The reporting follows the guidelines of the Consolidated Criteria for Reporting Qualitative Research (COREQ) [23].

## Results

### Sentiment Analysis

Overall, 148,541 tweets were passed to the sentiment analyser after pre-processing. Table 1 outlines the sentiment distribution using VADER, TextBlob, and Flair, giving the percentage of positive, negative and neutral tweets identified in the dataset.

**Table 1:** Analysis results of VADER, TextBlob and Flair

Models	Positive	Negative	Neutral
VADER	55,040 (37.05%)	44,395 (29.89%)	49,106 (33.06%)
TextBlob	70,900 (47.73%)	22,729 (15.30%)	54,912 (36.97%)
Flair	31,389 (21.13%)	117,152 (78.87%)	0 (0.00%)

**Fig. 4:**
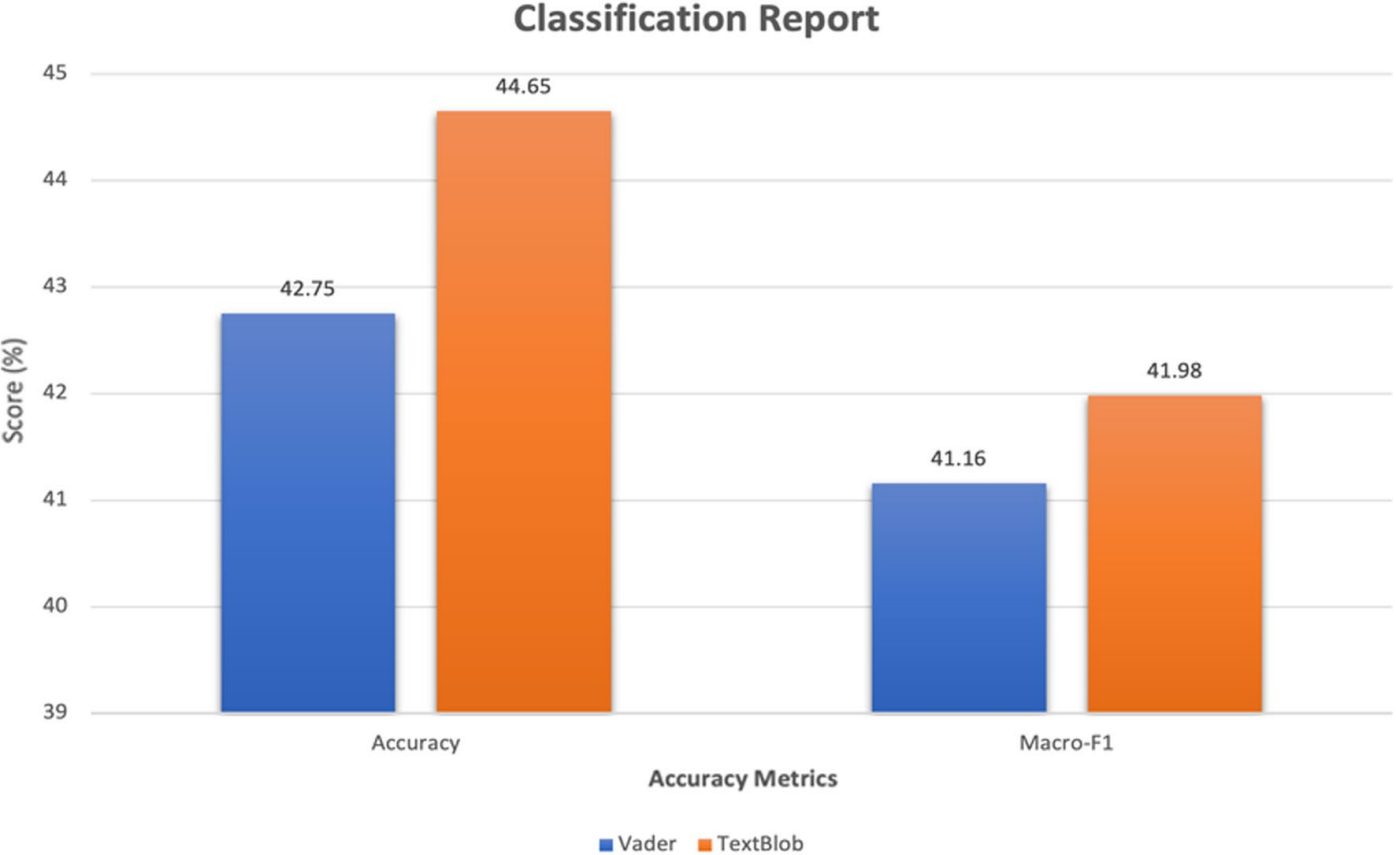
Classification report of VADER and TextBlob

The tweets collected from Twitter API’s are raw, meaning they are unlabelled and have unbalanced sentiment distribution. Hence, we annotated a random sample of approximately 1% of extracted tweets to evaluate the models used for performing sentiment analysis. The accuracy and macro-F1 score for the analysers are shown in Fig. 4.

**Fig. 5:**
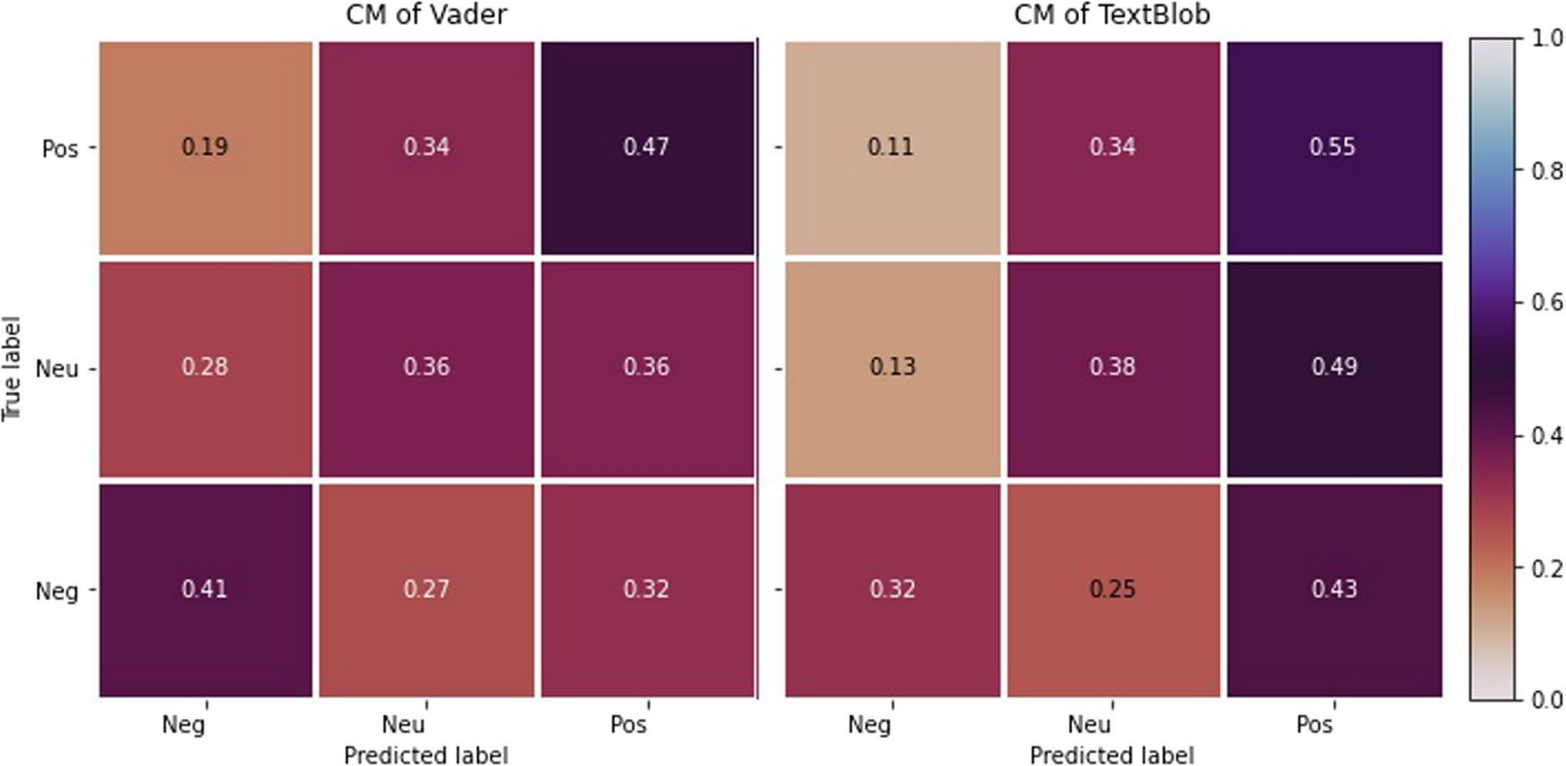
Confusion Matrices for VADER and TextBlob

The confusion matrix is plotted for two classifiers, as shown in Fig. 5 The cells along the anti-diagonal line show the percentage of tweets correctly predicted by the classifiers.

The classifiers have performed fairly alike, though TextBlob shows slightly better performance for positive tweets, whereas VADER for negative tweets in Fig. 4. The sentiments are spread across the matrix, showing the confused state of the models. This indicates that people had conflicting thoughts regarding Monkeypox spread and vaccine distribution. This is in line with the manual observation we did on the tweets.

### Content Analysis

We organized our data using ten main themes and eighteen issues as per the conceptual framework. Tables 2 and 3 provides an overview of our themes, issues and tweets explored therein.

**Table 2:** Tweets related to affordability, appropriateness, availability and accommodation

Determinants	Issue	Tweets
Availability and accommodation	Inaccessible vaccines	“Expanded vaccine supply will enable @nycHealthy; @_DCHealth to resume vaccination – which was cut short when limited initial supply was snapped up immediately; will allow other cities with #monkeypox outbreaks to start”
	Mass vaccination	“With monkeypox going around, why not have mass vaccination efforts for smallpox? This isn’t like COVID, we already have treatments and vaccines for monkeypox”
	Fear expressed by general public due to the priority-based vaccination format	“That information. Esp in a state that is about to roll back rights for the LGBTQA community”
Affordability	Indirect costs of vaccination	“I gotta find a way to Toronto so I can get the monkeypox vaccine. my public health region currently has no plan to do clinics and they best they said was to travel and spend the night in the city”
	Profit-making tactics by big companies	“As Questions Swirl Around Monkeypox Origins and Risk, Vaccine Makers Set Sights on Profits” “Coincidence? Monkeypox Arrives Just In Time To "Save" 2 Smallpox Vaccine Companies”
Appropriateness	COVID-19 vaccination references	“Don't get vaccinated. I can't be the only one who realizes monkeypox wasn't a problem before the covid vaccine”
	Lack of research claims	“I am withholding my concerns until more research is completed. Study raises concerns about the effectiveness of the monkeypox vaccine”
	Vaccine side effects	“That vaccine, however, can have harsh side effects, some of which can be life-threatening for immuno-compromised people, pregnant women and older adults”

**Table 3:** Tweets related to the ability to seek, perceive, reach, pay and engage

Determinants	Issue	Tweets
Ability to perceive	Mistrust	“I got the Monkeypox vaccine. Now I’m having thoughts that the government is conspiring to kill all gay people with it”
	Negligence	“Monkeypox, Hepatitis & Covid-19: Public Health has been Hijacked Big lie comes, there are no the virus named Monkeypox” “I will not take their vaccine or wear a mask”
	Confusion	“I am trying to learn about how to get a monkeypox vaccine but can't find any information on your website. Can you please share when or how you'll begin providing it?”
Ability to seeks	Labelling	“So far, monkeypox is not spreading within other groups besides gay & bi men. Monkeypox vaccination is not indicated for, or being made available to, the general population”
	Fear of being labelled	“They feared unintended consequences: heterosexual people assuming they’re not susceptible, closeted men avoiding vaccination, so they’re not seen as gay, and critics exploiting the infections to sow bigotry”
	Supporting vaccination	“I’m fortunate to have received my first dose of the Jynnos monkeypox vaccine”
Ability to reach	Poor management and distribution	“If rich countries hoard monkeypox vaccines, the fallout could threaten nascent efforts to rebuild global cooperation around future pandemics after the stark inequities of COVID-19 The same thing is happening right now with monkeypox. We have a vaccine stockpile yet sending none of it to the source”
	Long waiting period	“I finally was able to schedule a 2nd monkeypox vaccine two months after my first one, and after haggling with the website for 40 min that kept crashing and after four months of reporting and abstinence and talking about MPX, I burst into tears of frustration and rage and relief”
	Dismissing general population	“Finding a #monkeypox vaccine in the Bay Area is not easy right now. But there are still providers in the area offering the vaccine to eligible residents, including those without health insurance.” “We'll keep updating our guide as information changes” “Not me crying bc I’m ‘not eligible’ for a monkeypox vaccine”
Ability to pay	Profit making opportunity	“What If………. Vaccine makers made the disease they have a vaccine for; it would be a great scheme” “Curious, who stands to profit from the monkeypox vaccine?”
	Subsidized vaccines	“@nycHealthy stood up a free clinic for eligible New Yorkers to get a two-dose Monkeypox vaccine. A great call on their part right before NY Pride”
Ability to engage	Concern regarding vaccine	“There already exists a vaccine against monkeypox, but if this is a lab escape, it may be weaponized.” “Even More Cases Of The Monkeypox Have Been Confirmed As This Sickening Plague Continues To Spread. I wonder if the vaccine is allowing the spread, have they looked at it”
	Old generations do not require vaccines	“Just to let you know all the people who were vaccinated in the early 60’s against smallpox don’t need your monkeypox vaccine.” “It was incremental, building on success. I have the smallpox vaccine, which provides herd immunity to monkeypox. The new generation has neither memory of disease nor immunity. I have both. New generations believed disinformation because they lack experience/knowledge”

### Approachability

With the release of a vaccine to combat the mpox virus, social media users have expressed their desire for accurate information regarding the administration criteria and processes. Media outlets and news organisations disseminated information about the vaccination measures administered throughout regions, addressing people perspectives. Public health agencies have issued protocols and guidelines about the same topic. There has also been mention of the vaccination administration hierarchy depending on priority (Table 2).

### Acceptability

Due to the assumption that the virus is exclusively susceptible to members of the LGBTQ + community, the general population is hesitant to receive the vaccine for fear of being labelled as a member of the aforementioned group [24]. Along similar lines are also noticed homophobic and discriminatory attitudes. Both the LGBTQ + population and the non-vulnerable population have offered their diverse perspectives and experiences on the vaccine. Scars and other side effects of vaccination have been discussed. Others value the larger good of vaccine efficacy over their personal discomfort with the scars. For example, a tweet wrote, “If I'm not gay or whatever woke word of the day, then why should I get a vaccine for a rare virus mostly in Africa” (Table 2).

### Availability and Accommodation

Vaccinations for Smallpox are currently being offered for the mpox virus due to its 85% success rate [25]. With this update, the vaccine's availability has been called into question. Numerous individuals have expressed discontent with the availability and accessibility of vaccines. Contradictory perspectives and conversations on mass vaccinations are on the rise. The general public is anxious for their safety because immunisation facilities are prioritised for the most vulnerable population members and health care professionals (Table 2).

### Affordability

Although there have been no direct expenses associated with vaccination, there have been numerous cases of indirect costs incurred by vaccine recipients. Long-distance vaccination travel has imposed a costly strain on individuals. The original release of vaccines gave rise to rumours that the vaccine was designed to maximise profits (Table 2).

### Appropriateness

Multiple theories on the virus have arisen since the outbreak. People have begun to believe that the entire mpox situation is probable side effect of covid vaccines. There are claims that the vaccine can't be trusted since there hasn’t been enough research on smallpox vaccinations working against monkeypox (Table 2).

### Ability to Perceive

The general public has expressed scepticism over the health care measures implemented, including vaccination, in the previous few years due to all the health mishap that has occurred. A portion of the population is indifferent to the entire issue, disregarding the healthcare procedures and tweeting that the outbreaks are irrelevant. Questions regarding the necessity of vaccination, the duration of its efficacy, and the immunisation process are causing confusion among the general population (Table 3).

### Ability to Seek

The misinformation and labelling surrounding the virus have affected the opinions people possess on vaccination. While one part of the population has been claiming that there is no necessity for public vaccination since only the LGBTQ + community is susceptible to the virus, the other part fears the labelling they would receive if they do get vaccinated. Despite the stigma surrounding the vaccine, a part of the population is spreading the word against this misinformation and supporting vaccination procedures. The LGBTQ + community members have shared their experiences and have expressed acceptance through their tweets (Table 3).

### Ability to Reach

The power of the vaccine to reach people around the world is affected by different agents, such as transport and mobility. Poor management, as well as the poor distribution method of vaccines, have drastically affected the ability to reach people. The long waiting period and lack of vaccines have caused further issues to reach the public. There is also a lack of availability of vaccine guidelines. Priority vaccination has created eligibility criteria that dismiss the general population who also need vaccines (Table 3).

### Ability to Pay

The portion of the population that rejects the vaccine argues that vaccination is a money-making scheme, out of the many contradictory views on the subject. On the other hand, health officials and government bodies are taking measures to subsidize vaccine costs and related healthcare measures to encourage the public to accept Monkeypox vaccines (Table 3).

### Ability to Engage

Since the disclosure of the vaccination availability, varied responses from the public have been observed. While a considerable amount of the population was relieved and satisfied with this information, many people expressed their doubts with regard to the reliability of the vaccine. A set of people, especially the older generation, have been claiming their safety against the virus already, as they had taken doses of smallpox vaccination during the smallpox outbreak decades ago (Table 3).

## Discussion

**Fig. 6:**
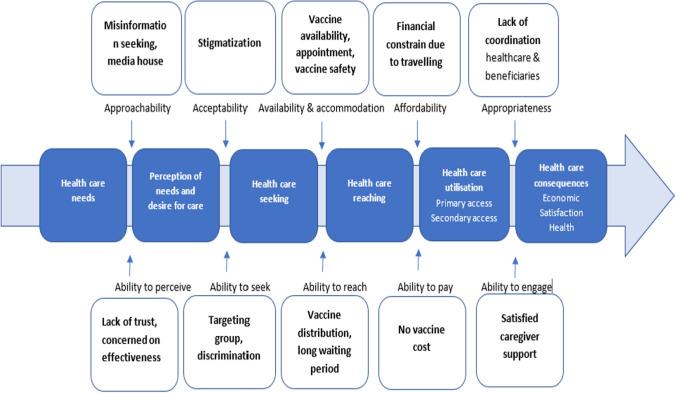
Adapted Levesque conceptual framework for healthcare

By the end of 2022, the number of mpox cases was on the decline; however, the virus has been reported from more countries than ever [26]. The situation have become a Public Health Emergency of International Concern, and in the time ahead, there would be a risk of outbreaks and the emergence of diseases. As it was decleared as a situation of public health emergency of international concern there is a risk of outbreak and emergence of diseases in future. The situation calls for improved planning and reaction to mpox. Therefore, adopting preventive measures, developing a targeted vaccination strategy, tailored communication strategy, and ensuring high uptake, as and when needed, require advanced planning. The health systems have initiated a swift response by delivering various health services, including vaccination, to curb the escalating outbreak. However, multiple factors have impacted access to healthcare services, particularly vaccination. This paper has highlighted how the process of access to vaccination is influenced, how the healthcare agencies have adopted immediate measures to respond to the ongoing mpox outbreak, and how the factors have affected the dissemination of scientific information using the “Levesque conceptual framework for healthcare access” [18]. This model of accessing the vaccines is evident in this study’s findings in the Fig. 6.

As the mpox surge pattern shows, there was a cascading impact across the LGBTQ + populations, which caused an impression mark among the community as the spreader. Similar to this study and as emphasized by this model, the stigma associated with human immunodeficiency virus (HIV) infection is still well recognized as a disease that occurs between men who have sex with men and within communities of the LGBTQ + community [27]. People sought credible information regarding the vaccination's criteria and processes for administration [6]. However, due to the dissemination of false information, some questioned the accessibility and uptake of the vaccines. Even the Coronavirus Disease 2019 (COVID-19) vaccination rate dropped as a result of the negative effect of misinformation on vaccination [28]. It has been noted that there was a priority-based hierarchy for administering the vaccine as an immediate move. The World Health Organization (WHO) and other studies have highlighted and supported a priority-based hierarchy for vaccination [29, 30].

The level of knowledge of the general population and physicians concerning monkeypox and related vaccinations is crucial for achieving high vaccination coverage and making informed policy decisions [31, 32]. It is imperative to acknowledge that insufficient knowledge among physicians may hinder efforts to detect and manage cases of monkeypox and establish policies that promote national vaccination strategies [32]. Additionally, a lack of awareness among the general population may serve as a potential basis for addressing vaccine hesitancy and lower vaccine uptake [31]. Therefore, policymakers must prioritize disseminating adequate information to both groups to ensure optimal vaccination coverage and inform policy decision-making.

To an immediate measure and effective utilization of resources and judicious use of the vaccines, various interim guidance on vaccines and immunization for monkeypox can be adopted [33]. Due to the misperception and stigma that the virus is exclusively susceptible to members of the LGBTQ + community, the general population is hesitant to receive the vaccine for fear of being labelled as members of the aforementioned group. These constraints on access have also been noted for other stigmatised disorders [34]. It have been reported that monkeypox vaccination acceptance in all participants was 56.0% [35]. As a result of the initial outbreak affecting the LGBTQ + community disproportionately, issues of homophobia and stigmatisation arose, resulting in the incorrect labelling of monkeypox as the “LGBTQ + community Man Disease”. Similar accusations have been made throughout HIV or Acquired immunodeficiency syndrome (AIDS) epidemics [36].

Stigmatization-driven mental health impact is well documented among the LGBTQ + community, which directly impacts accessing healthcare, and to cope with that, implementation of the comprehensive Mental Health Action Plan 2013–2030 necessitates strengthening the mental health status among the community [37]. The vaccine’s accessibility has also been questioned by the general population. Similar issues have been reported during the recent COVID-19 pandemic [38]. Many people have voiced their displeasure with the accessibility and availability of vaccines similar to the recent COVID-19 pandemic [39].

For the interrupted logistic supply and to achieve successful vaccine administration, nations should adopt strategies that can bridge this lacuna. Health officials made an effort to provide the vaccination free of cost to the large gathering of high-risk individuals, but this resulted in an uneven distribution of the vaccine to the general public. Despite the absence of direct costs associated with vaccination, there have been countless instances in which persons who required the vaccine have incurred indirect costs. Because there are no local vaccination facilities, certain individuals are forced to travel a considerable distance to acquire vaccinations, which has impacted vaccination coverage. Since the smallpox vaccination is somewhat effective, it is currently used to prevent monkeypox, and questions have been raised about its efficacy, duration, and inequity. Similar to these instances, antimalarial medications were also used to treat other diseases [40, 41]. Concerns have been expressed by the general population regarding the adopted healthcare measures, particularly vaccination. Creating communication strategies or hosting workshops on communication and trust-building activities connected to vaccines and immunisation, immunisation advisory bodies, etc. can assist in alleviating these undesirable fears [42].

Various factors, such as transportation and mobility, have an impact on the vaccine’s capacity to reach individuals worldwide. The capacity to reach people has been significantly impacted by inefficient management and immunisation delivery methods. Among the numerous contradictory opinions regarding the vaccine, the fraction of the population that rejects the vaccine is seen as meaning that independent enterprises, the pharmaceutical industry, and other organisations consider the vaccine rollout as a profit-making opportunity. To persuade the public to take Monkeypox vaccines, health officials and government organisations are subsidising vaccine costs and accompanying healthcare measures. Since the public's awareness of the availability of vaccinations, various reactions have been recorded.

While a considerable amount of the population was relieved and satisfied with this information, many people expressed their doubts concerning the reliability of the vaccine. As a result of receiving smallpox vaccinations during the smallpox pandemic decades ago, a group of individuals, particularly the older generation, have already asserted their immunity to the virus. To manage and reduce these challenges, a balance must be achieved. It provides critical insights into overcoming these gaps in managerial and organisational operations, professional conduct, and policy-making processes [43]. Using all the information available, countries should work to create plans for the judicious use of the monkeypox vaccines that are currently available.

Due to the stigmatisation of the virus and the widespread assumption that it primarily affects the LGBTQ + community, people have shown hesitation to get vaccinated. They are wary of doing so out of concern that they would be associated with the stigmatised group. These access limitations have also been noted for other stigmatised disorders [34]. Due to the fact that the mpox vaccine is an adult vaccination, implementation would require an in-depth understanding of its technical components, as adult immunisation is a topic that the majority of Low-and-middle income countries (LMIC) must research and make informed judgements [44]. Adult vaccination has not always been seen as a highly technical subject, as indicated by the fact that it was not included in India’s first-ever national vaccine policy in 2011 [45]. The National Technical Advisory Group on Immunization in India has rarely considered adult immunisation, although NTAGI lacks a clear mandate to provide recommendations about adult vaccinations beyond universal immunization programme (UIP) [46]. Despite formal recommendations from several high-income nations, adult vaccination rates are still low, despite relatively better coverage in high-risk populations.

There have been reports of vaccine hesitancy in light of the mpox outbreak, underscoring the necessity for a global policy [3]. It has been observed that the vaccine utilized for mpox is predominantly accessible in industrialized nations. A few instances of mpox have been reported in the Group of Twenty (G20) countries, where the vaccine is still unavailable due to lack of accessibility. For instance, Mexico recorded 3928 confirmed cases but lacked vaccine availability [47, 48] To address this situation, Mexican activists have called for urgent measures to provide the vaccine [49].

Although mpox was first discovered in Africa, it has since spread to non-endemic countries. It will have a detrimental effect as the outbreak spreads to non-endemic countries where the vaccine is unavailable [10]. The inequitable distribution of the mpox vaccine is evident, and it is primarily affected in countries with low economic conditions. To mitigate that, the proposed strategy suggested by Africa and India in response to crucial shortages and breakdown of critical supply chains in combating the COVID-19 pandemic, South Africa and India proposed a waiver of certain provisions of the Trade-Related Intellectual Property Rights (TRIPS) waiver agreement on October 2020 to the TRIPS waiver Council at the World Trade Organization (WTO) to diversify the manufacturing of supplies may be adopted [50]. However, on the other hand, multiple nations opposed the waiver.

### Strengths and Limitations

The present study has several scientific strengths and limitations that provide valuable insights into current attitudes and potential strategies for addressing concerns. Firstly, the study collected a large sample from multiple countries using Twitter. This approach allowed for the collection of near real-time data, providing timely and relevant information for analysis. Because of the diverse nature of the data as it can be generalized to the broader population since it includes users from various demographic and geographic backgrounds. Additionally, Twitter data is readily available and accessible, which reduces the cost of data collection and analysis compared to traditional methods like surveys and interviews.

The current investigation is accompanied by several constraints, which may provide avenues for future researchers to explore. Firstly, future studies can aim to increase the representativeness of the sample of Twitter users, as not all individuals have equal access to or utilize Twitter. Secondly, researchers can develop methods to address the dissemination of false information and the potential influence of automated bots on users’ attitudes towards vaccination. Lastly, future studies can expand their focus to include data from individuals who communicate in languages other than English, which may provide a more comprehensive understanding of the topic. Overall, this investigation highlights essential avenues for future research in this area.

## Conclusion

The success of adult immunization requires a robust communication strategy. As the need and demand for adult vaccination will increase in the time ahead, the government policy needs to maximise uptake and reduce infection.

## Data Availability

The data can be accessed from three authors: PR, VSD, and CK. This document confirms that each author in the manuscript had complete access to the study's data and accepted responsibility for the study’s publication. Data will be made available on request for publication purposes on acceptance of the manuscript in a journal.

## Abbreviations

AIDS: Acquired immunodeficiency syndrome
API: Application Programming Interface
COREQ: Consolidated Criteria for Reporting Qualitative Research
COVID-19: Coronavirus Disease 2019
G20: Group of Twenty
HIV: Human immunodeficiency virus
LGBTQ +: Lesbian, Gay, Bisexual, Transgender, and Queer
LMIC: Low-and-middle income countries
mpox: Monkeypox
MSM: Men who have sex with men
NTAGI: National Technical Advisory Group on Immunization
PHEIC: Public health emergency of international concern
TRIPS: Trade-Related Intellectual Property Rights
UIP: Universal immunization programme
VADER: Valence Aware Dictionary and Sentiment Reason
WHO: World Health Organization
WTO: World Trade Organization
